# Assessment of Antiobesity Potential of *Achyranthes aspera* Linn. Seed

**DOI:** 10.1155/2012/715912

**Published:** 2012-06-27

**Authors:** Neerja Rani, Surendra Kumar Sharma, Neeru Vasudeva

**Affiliations:** Department of Pharmaceutical Sciences, Guru Jambheshwar University of Science and Technology, Hisar, Haryana 125001, India

## Abstract

The present study was designed to evaluate the quality control parameters, quantitative phytochemical analysis (total phenols, total flavonoids, and total saponin content), and the antiobesity effect of ethanol extract of *Achyranthes aspera* Linn. seed (EAA) by employing *in vitro* and *in vivo* models. In *in vitro* study, the inhibitory activity of EAA on pancreatic amylase and lipase was measured. The *in vivo* pancreatic lipase activity was evaluated by measurement of plasma triacylglycerol levels after oral administration of EAA along with lipid emulsion to Swiss albino mice. The EAA inhibited pancreatic amylase and lipase activity in vitro and elevations of plasma triacylglycerol level in mice. Furthermore, the antiobesity effect of EAA (900 mg/kg) was assessed in mice fed a high-fat diet with or without EAA for 6 weeks. EAA significantly suppressed the increase in body, *retroperitoneal adipose* tissue, liver weights, and serum parameters, namely; total cholesterol, total triglyceride, and LDL-cholesterol level. The anti obesity effects of EAA in high-fat-diet-treated mice may be partly mediated through delaying the intestinal absorption of dietary fat by inhibiting pancreatic amylase and lipase activity. Histopathological effects of EAA on the liver of mice were also assessed.

## 1. Introduction

 Obesity has become one of the fastest growing major disorders throughout the world [1, 2]. Many factors have been attributed to an epidemic of obesity including sedentary lifestyle, high-fat diets, and consumption of modern fast foods [3]. Obesity leads to hypertension, diabetes, myocardial infarction, and peripheral vascular disease. Obesity therapies include reducing nutrient absorption and applying anorectic drugs, thermogenic drugs, or drugs that affect lipid mobilization and utilization. A long history of medicinal use of natural products exists in the management of obesity and hyperlipidemia [4]. They play a principle role in the introduction of new therapeutic agents [5, 6]. Development of inhibitors of nutrient digestion and absorption, which reduce energy intake through gastrointestinal mechanism without altering any central mechanisms, is one of the most important methods in the treatment of obesity. At present, the potential of natural products for the treatment of obesity is still largely unexplored and might be an excellent alternative strategy for the development of safe and effective antiobesity drugs [7].

Naturally occurring phytochemicals present an opportunity for the discovery of newer antiobesity agents. Literature reports various digestive enzyme inhibitors isolated from plants, namely; crude saponins from *Platycodi radix* [8], ginseng saponin [9], tea saponin [10], licochalcone A from roots of *Glycyrrhiza uralensis* [11], platycodin D from fresh roots of *Platycodon grandiflorum* [12], dioscin from *Dioscorea nipponica* [13], and phenolic constituents from the leaves of *Nelumbo nucifera *[14]. *Achyranthes aspera* seeds are also reported to contain oleanene-type triterpenoid saponins [15, 16]. However, the antiobesity effect of seeds of  *Achyranthes aspera *has not been examined yet. Therefore, present study was focused on the quantification of phytoconstituents and also *in vitro* and *in vivo* antiobesity evaluation of *Achyranthes aspera* Linn. seed.

## 2. Materials and Methods 

### 2.1. Chemicals

Pancreatic *α* amylase, pancreatic lipase, and glycyrrhizic acid were purchased from Sigma (Aldrich Co., St. Louis, MO, USA). Methanol (HPLC grade), casein, soluble starch, vitamin and mineral mixture, glyceryl trioleate, lecithin, sodium cholate, and TES buffer were purchased from Hi-media. All other chemicals were of reagent grade.

### 2.2. Animals

Male Swiss albino mice (5 weeks old) were used for the *in vivo* models. The animals were housed for 1 week under a 12 h/12 h light/dark cycle in a temperature humidity-controlled room. The animals were given free access to food and water. After adaptation to the lighting conditions for 1 week, the healthy animals were used in the *in vivo* models. The experimental protocols were approved by the Institutional Animal Ethical Committee, Guru Jambheshwar University of Science and Technology, Hisar, India (Regn no. 0436).

### 2.3. Plant Material

The seeds of *Achyranthes aspera *Linn. (Amaranthaceae) were collected from the campus of Guru Jambheshwar University of Science and Technology, Hisar in the month of April, 2010. The plant was taxonomically identified and authenticated by Dr. H. B. Singh, Head, Raw Materials Herbarium and Museum Division of National Institute of Science Communication and Information Resources. The voucher specimen (Pg-10-01) has been deposited in the herbarium section of the Pharmacognosy Division, Department of Pharmaceutical Sciences, Guru Jambheshwar University of Science and Technology, Hisar for further reference.

### 2.4. Preparation of Extract

The powdered seed (1 kg) was defatted with petroleum ether (60–80) and then extractedwith 95% ethanol (4 L) by continuous soxhlation process for 7 days. The extract was filtered and concentrated to dryness using a rotary evaporator. The percentage yield of chocolate-brown-coloured extract was found to be 2.5%. Dried extract was stored at 4°C till further use.

### 2.5. Quality Control Parameters of EAA

The EAA was subjected to various quality control parameters according to the Indian Pharmacopoeia [17] and WHO Guidelines [18]. The phsyco-chemical parameters, namely, ash values, loss on drying, and heamolytic activity; heavy metal analysis (Lead, Cadmium, Arsenic), microbial (*E. coli*, *Salmonella sp., S. aureus*), and aflatoxin (B1 + B2 + G1 + G2) contamination were determined. The preliminary phytochemical screening was also performed for the presence of major phytochemical constituents in EAA according to standard methods [19].

### 2.6. Determination of Total Phenol Content

The total phenol content of EAA was estimated using the Folin-Ciocalteu method adapted from Singleton and Rossi [20]. EAA (0.1 mL, 1 mg/mL) was oxidized with 0.25 mL of 10% (v/v) Folin-Ciocalteu's reagent and neutralized by adding 1.25 mL of 20% sodium carbonate. The absorbance was measured at 685 nm after incubating at 40°C for 40 min. Results are expressed as mg/g of gallic acid.

### 2.7. Determination of Total Flavonoid Content

The AlCI_3_ method adapted from Lamaison and Carnet [21] was used for the determination of the total flavonoid content of the EAA. 0.4 mL (10 mg/mL) of EAA was added to 2 mL of a solution of 2% AlCI_3_·6H_2_O. After proper mixing, the mixture was incubated for 10 min at ambient temperature. The absorbance of the solution was read at 440 nm. Flavonoid contents are expressed in mg/g of quercetin.

### 2.8. Determination of Total Saponin Content

For total saponin content determination 2 g of EAA was blended with 2 mL of concentrated NH_4_OH (37%) for 3 min. The pH of solution was adjusted to a pH 7.0 with H_3_PO_4_ and then 1 mL of 10% diastase was added. This was incubated at 37°C for 30 min, cooled to room temperature, and transferred to a 100 mL volumetric flask with CH_3_OH. The final extract was diluted to volume with additional CH_3_OH and filtered through Whatman no. 42 paper prior to analysis [22]. Before injection to HPLC column, extracts were filtered through a 0.45 *μ*m membrane filter (Millipore, Bedford, USA).

Chromatographic analysis were carried out on the HPLC system (Agilent Technologies 1260 infinity) and consisted of 1260 DVD-VL/1260 ALS/1260 Binary pump and UV/visible detector. Separation of saponins was done using a Zorbax Eclipse XDB-C18 (Analytical 4.6 × 250 mm ID, particle size 5 *μ*m) column at 1.5 mL/min flow rate. Detection was made at 245 nm at 25°C. The analysis used 20 *μ*L of a sample solution. The mobile phase consisted of methanol, water, and acetic acid in the ratio of (60 : 34 : 6 v/v). The solvents were filtered and degassed prior to use. The glycyrrhizic acid was used as standard. Quantification of the saponin is expressed in *μ*g/g of EAA and determined by a standard curve from a plot of the peak area and matching concentration of the standard solution.

### 2.9. Measurement of *α*-Amylase Inhibitory Activity

The activity was measured using the method reported by Xiao et al. [23] and Yoshikawa et al. [24] with slight modifications. Substrate solution was prepared by dissolving soluble starch (500 mg) in 25 mL of 0.4 M NaOH and heating for 5 min at 100°C. After cooling in ice H_2_O, the pH of solution was adjusted to 7 with 2 M HCl, and water was added to adjust the volume to 100 mL. Sample solutions were prepared by dissolving EAA in acetate buffer (pH 6.5) to make 1, 2, 3, 4, and 5 mg/mL solutions. The substrate (40 *μ*L) and sample (20 *μ*L) solutions were mixed in a microplate well, and the mixtures were preincubated at 37°C for 3 min. Then 20 *μ*L of *α*-amylase solution (50 *μ*g/mL) was added to each well, and the plate was incubated for 15 min. The reaction was terminated by adding of 80 *μ*L of 0.1 M HCL; then 200 *μ*L of 1 mM iodine solution was added. The absorbances were measured at 650 nm. Inhibitory activity (%) was calculated as follows:

(1) Inhibition(%)  ={1−(Abs2−Abs1)/(Abs4−Abs3)×100},

where Abs1 is the absorbance of incubated solution containing EAA, starch and amylase, Abs2 is the absorbance of incubated solution containing EAA and starch, Abs3 is the absorbance of incubated solution containing starch and amylase, and Abs4 is the absorbance of incubated solution containing starch.

### 2.10. Measurement of Pancreatic Lipase Inhibitory Activity

Lipase inhibitory activity was measured according to the method of Han et al. [25] with slight modifications. Substrate solution was prepared by sonication (10 min in an ice bath) of a mixture of glyceryl trioleate (80 mg), lecithin (10 mg), and sodium cholate (5 mg) suspended in 9 mL of 0.1 M TES buffer (pH 7.0). EAA was dissolved in 0.1 M TES buffer to make 1, 2, 3, 4, and 5 mg/mL solutions. The substrate (20 *μ*L) and sample solutions (20 *μ*L) in microplate wells were preincubated for 3 min; then 10 *μ*L of lipase solution (20 *μ*g/mL) was added to each reaction mixture and incubated for 30 min at 37°C. The absorbance was measured at 550 nm using a microplate reader. Inhibitory activity (%) was calculated as follows:

(2) Inhibition(%)  ={1−(Abs6−Abs5)/(Abs8−Abs7)×100},

where Abs5 is the absorbance of incubated solution containing EAA, substrate, and lipase; Abs6 is the absorbance of incubated solution containing EAA and substrate; Abs7 is the absorbance of incubated solution containing substrate and lipase; Abs8 is the absorbance of incubated solution containing substrate.

### 2.11. Plasma Triacylglycerol Level after Oral Administration of Lipid Emulsion to Mice

The animals were divided in two groups and deprived of food overnight. Test group was orally administered lipid emulsion (5 mL/kg) with EAA (900 mg/kg). Positive control group was given lipid emulsion alone. The oil emulsion was prepared with 7 mL of olive oil, 93 mg of cholic acid, and 7 mL of deionized water. Food was withheld during the test. Blood samples were collected from the ophthalmic venous plexus at 0, 1, 2, 3, 4, and 5 h using a heparinaized capillary tube and centrifuged at 6300 rpm for 10 min. Plasma triacylglycerol levels were measured using a commercial triglyceride assay kit (Erba diagnostics).

### 2.12. High-Fat Diet-Induced Obesity

Male Swiss albino mice (5 weeks old) were acclimatized for 1 week fed a high-fat diet (HFD) for 2 weeks and randomLy divided into three groups matched for body weight [26]. Each group contained six animals. Control group was fed normal diet *ad libitum*. Test group received EAA (900 mg/kg) and high-fat diet. Positive control group received high-fat diet. The treatment was done for 6 weeks. The composition of high-fat diet was as follows (g/100 g food): corn starch, 10; sugar, 10; lard, 40; vitamin mixture, 1; mineral mixture, 4; casein, 20; cellulose, 5; soybean oil, 7; methionine, 3. The total food intake by each group was recorded at least twice weekly, and the body weight of each mouse was recorded once weekly. At the end of the experiment, the blood was taken by venous puncture under anesthesia with diethyl ether, and the mice were then killed with an overdose of diethyl ether. Experiments were performed in a ventilated room. The serum was prepared and frozen at −80°C until analysis. The liver and *retroperitoneal adipose* tissue were dissected and weighed. The glucose, triglyceride (TG), total cholesterol (TC), HDL cholesterol, and LDL cholesterol were measured using Triglyceride *E*-Test and Total Cholesterol *E*-Test kits. The atherogenic index was calculated as follows: total cholesterol−HDL cholesterol/ HDL cholesterol.

### 2.13. Statistical Analysis

The data were expressed as the mean ± SEM and were analysed by one-way ANOVA followed by Dunett's test; a *P* value of <0.05 is considered significant.

### 2.14. Histopathological Study

For histopathological studies livers of the sacrificed mice were dissected, removed, washed with normal saline, and put in 10% formalin solution. The fixed specimens were then trimmed, washed, and dehydrated in ascending grades of alcohol. The tissue specimens were cleared in xylene, embedded in paraffin, sectioned at 4–6 *μ* thickness, and stained with Hematoxylin and Eosin. Photomicrographs were obtained under compound trinocular microscope (Zeiss Primo star) according to Carleton [27].

## 3. Results

### 3.1. Quality Control Parameters of EAA

The physico-chemical parameters, namely, total ash, water soluble ash, and acid-insoluble ash were found to be 12.5, 5.5, and 3.65% w/w, respectively. The percentage moisture content was found to be 6.5% w/w. The haemolytic activity was found to be 2.0 units/g. The preliminary phytochemical screening of the EAA indicated the presence of mainly alkaloids, steroids, saponins, phenols, flavonoids, and fixed oils. The Atomic Absorption Spectroscopy study showed the presence of cadmium, lead, and arsenic in EAA but below the WHO permissible limits and therefore safe to use. EAA showed complete absence of *E. coli*,* Salmonella typhi*, and* Staphylococcus aureus*. Aflatoxin (B1 + B2 + G1+ G2) were found to be less than 5 ppb.

### 3.2. Total Phenol, Flavonoid, and Saponin Content

The total phenol content in EAA was found to be 0.34 mg/g in gallic acid equivalents. The content of flavonoids, in quercetin equivalents in mg/g of plant extract was found to be 0.30 mg/g. The concentration of total saponin found to be 147.7 *μ*g/g of EAA as quantified with HPLC. 

### 3.3. Effect of EAA on Pancreatic *α*-Amylase and Lipase Activity

**Figure 1:**
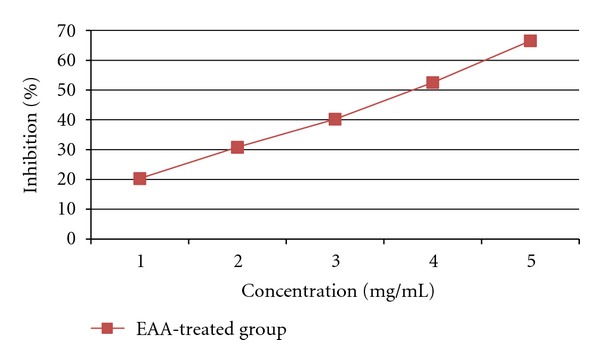
Inhibitory effect of EAA on *α*-amylase.

**Figure 2:**
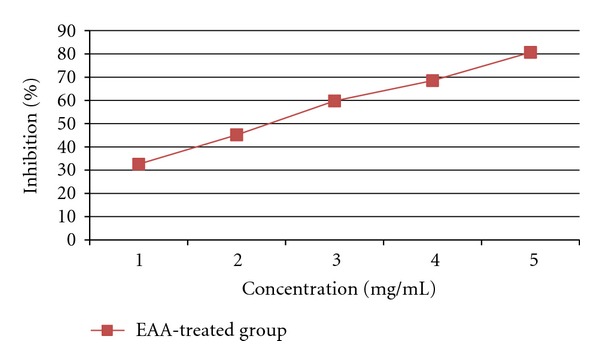
Inhibitory effect of EAA on lipase.

The inhibitory activity of EAA against pancreatic *α*-amylase and lipase was determined using different concentrations (1, 2, 3, 4, and 5 mg/mL). As shown in Figures 1 and 2 EAA inhibited the enzyme activities in a dose-dependent way. The inhibition of lipase by EAA (IC_50_ value; 2.34 mg/mL) was stronger than that of *α*-amylase (IC_50_ value; 3.83 mg/mL).

### 3.4. Effect of EAA on the Plasma Triacylglycerol Levels after Oral Administration of Lipid Emulsion to Mice

**Figure 3:**
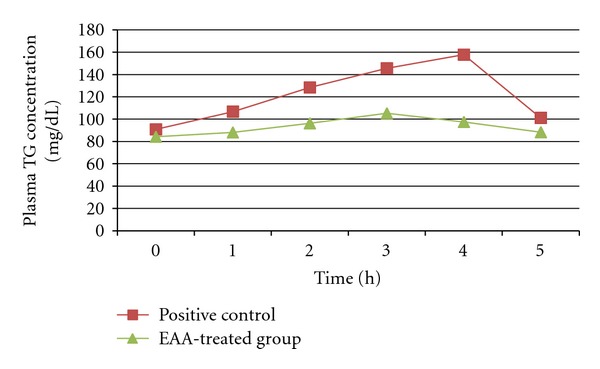
Effect of EAA on elevation of the plasma triacylglycerol (TG) level after oral administration of a lipid emulsion. Values are means ± SEM.

Figure 3 shows the serial changes in plasma triacylglycerol concentration when lipid emulsion with or without EAA was administered orally to mice. At 3 and 4 h after administration of EAA, the plasma triacylglycerol concentrations were significantly (*P* < 0.01) lower than those in the positive control group.

### 3.5. Effect of EAA on Food Consumption, Body, Retroperitoneal Adipose Tissue and Liver Weights, and Serum Parameters in Mice Fed a High-Fat Diet for 6 Weeks

The mean food consumption per week per mice was different between the control and high-fat diet groups throughout the whole experimental period, but it did not differ between the groups fed high-fat diet alone and high-fat diet plus EAA treated group, suggesting that the antiobesity effect of EAA was not mediated by a reduction of food intake (Table 1). The change in body weight of the groups during the experimental period of 6 weeks is shown in Table 1. The EAA significantly (*P* < 0.01) suppressed the body weight gain when compared to the control group fed on high-fat diet alone during experimental period. The oral administration of EAA to high-fat diet induced obese mice for 6 weeks cause significant reductions in retroperitoneal adipose tissues and liver weight as compared to high-fat diet.

The serum concentrations of TG, cholesterol, and LDL cholesterol were significantly lowered in the group fed high-fat diet and treated with EAA, than in the control group fed on the high-fat diet alone. Furthermore, the EAA increased the level of HDL cholesterol, leading to an improvement in the atherogenic index. There was no significant change in the glucose concentrations in the control, high-fat diet, and high-fat diet plus EAA-treated groups (Table 2).

### 3.6. Histopathological Study

**Figure 4:**
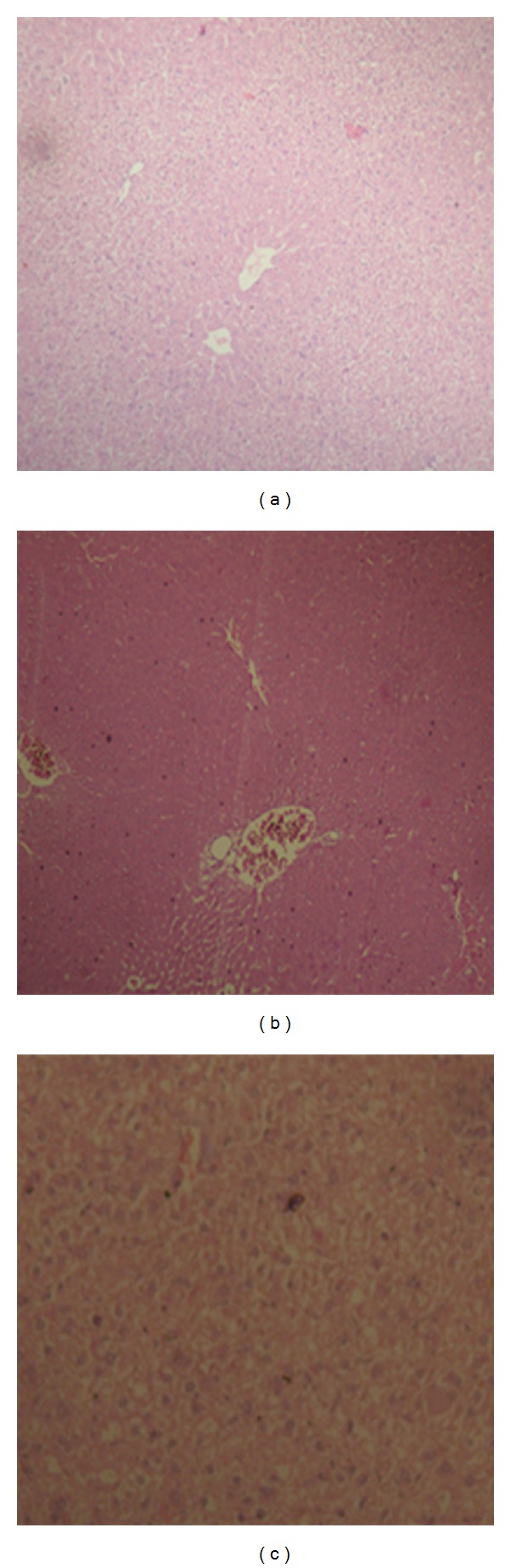
Effect of EAA on liver of mice (a) control (b) high-fat diet (c) EAA treated group.

Histopathological examination of liver of the control group mice fed on normal diet revealed normal histological picture of hepatic lobule which consists of central vein surrounded by normal hepatocytes (Figure 4(a)). Examination of liver of mice fed on high-fat diet showed fatty degeneration of hepatocytes and infiltration of leucocytes in hepatic sinusoid (Figure 4(b)). Liver of mice given orally EAA (900 mg/kg) showed marked improvement in fatty degeneration with no observed pathological lesions (Figure 4(c)). 

## 4. Discussion

Obesity is caused by excess caloric intake [28] and this can be improved by inhibiting pancreatic lipase activity and by inhibiting or delaying lipid absorption [29]. Inhibition of *α*-amylase activity and inhibition of carbohydrate absorption also play an important role in the prevention and treatment of diabetes [30]. **α*-*amylase, one of the digestive enzyme secreted from the pancreas and salivary glands, is involved in an important biological process such as digestion of carbohydrates. Many crude drugs inhibit**α*-*amylase activity [31]. Natural *α*-amylase inhibitors have been demonstrated to be beneficial in reducing postprandial hyperglycemia by slowing down the digestion of carbohydrates and, consequently the absorption of glucose. Reducing postprandial hyperglycemia prevents, glucose uptake into adipose tissue to inhibit synthesis and accumulation of triacylglycerol [32]. On the other hand, it is well known that dietary lipid is not directly absorbed from the intestine unless it has been subjected to the action of pancreatic lipase. The two main products formed by the hydrolysis of triglycerides by pancreatic lipase are fatty acid and 2-monoacylglycerol. Based on these facts, inhibition of these digestive enzymes is beneficial in treatment of obesity. In the present study, we performed *in vitro* and *in vivo* experiments to evaluate the effects of EAA on lipid and carbohydrate absorption. EAA inhibited *α*-amylase and lipase activities *in vitro* studies in dose-dependent manner. The inhibition of lipase by EAA (IC_50_ value; 2.34 mg/mL) was stronger than that of *α*-amylase (IC_50_ value; 3.83 mg/mL). The EAA has also significantly decreased postprandial triglyceride level at 3 and 4 h in *in vivo* studies at the dose of 900 mg/kg body weight after oral administration of olive oil in mice.

Further study demonstrated that EAA could improve high-fat-diet-induced obesity. The weight of whole body, food consumption, adipose tissue and liver weights, and serum parameters, namely, total cholesterol, total triglyceride, LDL cholesterol, HDL cholesterol, and glucose levels were determined in mice fed a normal, high-fat diet and the high-fat diet along with EAA. Although cumulative food intake over the experimental period was similar in all groups, the high-fat diet produced a marked increase in body weight after six weeks of feeding compared with normal diet group. This showed that high-fat diet group had higher energy intake than the normal diet group, and the high-fat diet contributed to migration of obesity [33–35]. Parallel to the change of body weight, the weights of adipose tissues and livers were higher in the high-fat diet group than in the normal diet group and in the high-fat diet along with EAA group. Long-term feeding of EEA to mice caused significant changes in serum parameters, namely, decreased levels of total cholesterol, total triglyceride, and LDL-cholesterol, but an increased HDL cholesterol level. There was no significant change in the glucose concentrations in the control, high-fat diet, and high-fat diet plus EAA-treated groups; this suggest, that obesity induced by the high-fat diet in mice did not cause diabetes.

Literature reports that saponins from natural products for example, crude saponins isolated from *Platycodi radix* [8], ginseng saponin [9], tea saponin [10], phenolic compounds [36], and flavonoids [37] showed strong antiobesity activity by inhibiting pancreatic lipase and suppressing the increase of body weight induced by a high-fat diet. *Achyranthes aspera* seeds are reported to contain of oleanene-type triterpenoid saponin [15, 16]. In our studies oleanene-type triterpenoid saponin was found to be 147.7 *μ*g/g of EEA along with 0.34 mg/g of phenols and 0.30 mg/g of flavonoids, which may be responsible for the antiobesity activity. 

In conclusion, *Achyranthes aspera *seed may prevent obesity by reducing the excess accumulation of body fat and changing the serum lipid profile.

## Figures and Tables

**Table 1:** Effect of EAA on the body weight, food intake, retroperitoneal fat, and liver weight in mice fed on the high-fat diet for 6 weeks.

Group	Body weight gain (g)	Total food intake (g)	Retroperitoneal fat (g)	Liver weight (g)
Control	2.81 ± 0.28	6.73 ± 0.10	0.98 ± 0.11	3.13 ± 0.11
HF diet	6.21 ± 0.75	7.20 ± 0.25	2.32 ± 0.37	4.45 ± 0.35
HF diet + EAA	1.66 ± 0.57**	7.07 ± 0.63	0.66 ± 0.21**	3.31 ± 0.19**

Data are expressed as mean ± SEM, *n* = 6, *P < 0.05, **P < 0.01 compared with high-fat diet group.

**Table 2:** Effect of EAA on the blood parameters in mice.

Serum parameter	Control	HF diet	HF diet + EAA
Glucose (mg/dL)	164.68 ± 3.03	151.78 ± 8.10	142.52 ± 1.56
Total triglyceride (mg/dL)	88.72 ± 3.02	125.62 ± 5.70	89.81 ± 3.29**
Total cholesterol (mg/dL)	132.23 ± 9.87	200.90 ± 17.34	138.87 ± 10.30**
HDL-cholesterol (mg/dL)	68.06 ± 5.33	43.88 ± 3.41	65.43 ± 4.65**
LDL-cholesterol (mg/dL)	56.16 ± 10.07	136.09 ± 15.97	58.48 ± 12.80**
Atherogenic index	1.02 ± 0.26	3.63 ± 0.40	1.18 ± 0.23**

Data are expressed as mean ± SEM, *n* = 6, **P < 0.01 compared with high-fat diet group.
